# NEWS2 versus a single-parameter system to identify critically ill medical patients in the emergency department

**DOI:** 10.1016/j.resplu.2020.100020

**Published:** 2020-08-06

**Authors:** Stine Engebretsen, Stig Tore Bogstrand, Dag Jacobsen, Valeria Vitelli, Rune Rimstad

**Affiliations:** a Emergency Department, Division of Emergencies and Critical Care, Oslo University Hospital, Postboks 4950 Nydalen, 0424, Oslo, Norway; b Institute of Clinical Medicine, University of Oslo, Postboks 1171 Blindern, 0318, Oslo, Norway; c Department of Forensic Sciences, Oslo University Hospital, Postboks 4950 Nydalen, 0424, Oslo, Norway; d Institute of Health and Society, University of Oslo, Postboks 1130 Blindern, 0318, Oslo, Norway; e Department of Acute Medicine, Division of Medicine, Oslo University Hospital, Postboks 4950 Nydalen, 0424, Oslo, Norway; f Oslo Center for Biostatistics and Epidemiology, Department of Biostatistics, University of Oslo, Postboks 1122 Blindern, 0317, Oslo, Norway; g Medicine, Health, Patient Safety and Integration, Oslo University Hospital, Postboks 4950 Nydalen, 0424, Oslo, Norway

**Keywords:** Emergency service, Hospital, Critical care, Hospital rapid response team, Early warning score, Internal medicine

## Abstract

**Aim:**

To test National Early Warning Score 2 (NEWS2) versus a single-parameter system to identify critically ill general medical patients in the emergency department (ED), by 1) testing NEWS2s prediction of and association with primary outcome ‘mortality’ (hospital or 30 day) and secondary outcomes ‘intensive care unit (ICU) admission’ and ‘critical care in ED’ and 2) comparing this for different NEWS2 cut-offs and the single-parameter system in use.

**Methods:**

Register-data on adult triage 1 and 2 patients with complete NEWS2 from 2015 and 2016 were retrieved. Prediction was assessed using area under the receiver-operating characteristic curve. Associations were analyzed using multiple logistic regression.

**Results:**

1586 patients were included. NEWS2 showed poor prediction of ‘mortality’ (AUC 0.686, CI 0.633–0.739) and adequate prediction of ‘ICU admission’ (AUC 0.716, CI 0.690–0.742) and ‘critical care in ED’ (AUC 0.756, CI 0.732–0.780). It was strongly associated with all outcomes (all p<0.001). All NEWS2 cut-offs and the single-parameter system showed poor prediction of all outcomes (all AUCs <0.7). The single-parameter system had the strongest association with ‘mortality’ (OR 1.688, CI 1.052–2.708, p<0.05) and ‘critical care in ED’ (OR 3.267, CI 2.490–4.286, p<0.001). NEWS2 > 4 had the strongest association with ‘ICU admission’ (OR 2.339, CI 1.742–3.141, p<0.001).

**Conclusion:**

For identification in order to trigger a response in the ED, outcomes closest in time seem most clinically relevant. As such, the single-parameter system had acceptable performance. NEWS2 > 4 should be considered as an additional trigger due to its association with ICU admission.

## Introduction

Critically ill medical patients in the Emergency Department (ED) are heterogeneous, and correct identification is essential to optimize initial management, ensure correct resource use and avoid adverse events.^1^ Identification and management seem to vary from ED to ED, as there, unlike some other patient groups,^2^^,^^3^ are no standard guidelines.^4^ Subgroups with specific diagnosis often have predefined pathways, but the majority is undiagnosed with more general signs and symptoms of organ failure.^1^ Although triage systems are used in most EDs to discriminate patients, these are often symptom-based and can be provider-dependent.^5^

Two types of trigger systems are used to identify deteriorating ward patients. Single-parameter systems use a predefined set of physiological parameters to call a medical emergency team. Early warning score systems (EWSS) allocate points to different levels of physiological parameters, and a total score of a preset level is used to call a critical care outreach team.^6^ Studies have found that the latter is better at discriminating patients at risk of unplanned intensive care (ICU) admission, cardiac arrest or death.^6^^,^^7^

One recent and much used EWSS is the National Early Warning Score (NEWS) and the updated NEWS2.^8^ Studies have found it better than other EWSSs and single-parameter systems at discriminating risk of adverse outcomes.^7^, ^8^, ^9^ It has also shown good performance in ED populations such as infection/sepsis patients,^10^, ^11^, ^12^ patients with respiratory illnesses^13^^,^^14^ elderly patients,^15^ and in general populations.^16^^,^^17^ It has, to our knowledge, not been tested on critically ill medical ED patients.

The aim of this study was thus to test the ability of NEWS2 to identify these patients, and compare it to the single-parameter system already in use in our hospital. More specific, the objectives were 1) to test NEWS2s prediction of and association with the primary outcome ‘mortality’ and secondary outcomes ‘ICU admission’ and ‘critical care in ED’, and 2) to compare this for different cut-offs of NEWS2 and the system in use.

## Methods

### Study setting

This register-based cohort study was performed at Oslo University Hospital (OUH) Ullevål, a tertiary hospital in Oslo with all sub-specialties in internal medicine. In 2015 the ED saw 28 ​000 patients; considered a large-volume ED in the Norwegian setting. The admittance rate was 90%, of which 50% were adult medical patients with a full range of medical conditions. Patients are referred to specialist care by primary care physicians or ambulance personnel, self-referral is rare. Referring of patients happens by telephone before arrival, and it is decided what specialty (ie medical, surgical, orthopedic or other) should review the patients in the ED. At the time of the study no emergency medicine specialty existed. Patients with conditions not needing specialist care in the hospital are seen by their General Practitioner or in Emergency Centers.

### Participants and data sources

Medical triage 1 and 2 patients were considered to be potentially critically ill. Both groups are referred to the medical specialties before arrival to the ED. Medical triage 1 patients are identified before arrival by ambulance personnel or at ED triage/later in the ED stay using a single-parameter system, the OUH-criteria (Table 1). They are managed by a multidisciplinary team in resuscitation rooms in the ED. Medical triage 2 patients, as well as lower triage-categories, are identified using Manchester Triage System (MTS). Medical triage 2 patients are seen by an ED nurse immediately after triage and by a medical doctor within 10 ​minutes from triage. All patients are observed according to triage category and NEWS2 score during the stay in ED. Myocardial infarction, cardiac arrest and stroke patients have predefined pathways, but are sometimes managed by the team or triaged using MTS.

**Table 1:** NEWS2 and OUH-criteria.

NEWS2								OUH-criteria
Points	3	2	1	0	1	2	3	
RR (per minute)	≤8		9–11	12–20		21–24	≥25	<8 or >40
SpO2 1 (%)	≤91	92–93	94–95	≥96				<85
SpO2 2∗ (%)	≤83	84–85	86–87	88-92 or	93-94 on	95–96	≥97 on	
				≥93 on air	O2	on O2	O2	
Air/O2		O2		Air				
SBP (mmHg)	≤90	91–100	101–110	111–219			≥220	<90
Pulse (per minute)	≤40		41–50	51–90	91–110	111–130	≥131	<35 or >130
Consciousness				Alert			CVPU	GCS <9
Temperature (°C)	≤35.0		35.1–36.0	36.1–38.0	38.1–39.0	≥39.1		<32

NEWS2: National Early Warning Score 2, OUH: Oslo University Hospital, RR: respiration rate, O2: oxygen, SBP: systolic blood pressure, CVPU: confusion, verbal, pain, unresponsive, GCS: Glasgow Coma Scale, ∗ For patients with hypercapnic respiratory failure.

Two registers, using routinely collected data from medical records, contained data about the patients. All medical triage 1 patients from 2015 and 2016, except those not holding a Norwegian social security number (n ​= ​44 in the study period), were included in and retrieved from the first register. The other register had data on every 5^th^ admitted medical triage 2 patients from the same period, these were also retrieved.

**Fig. 1:**
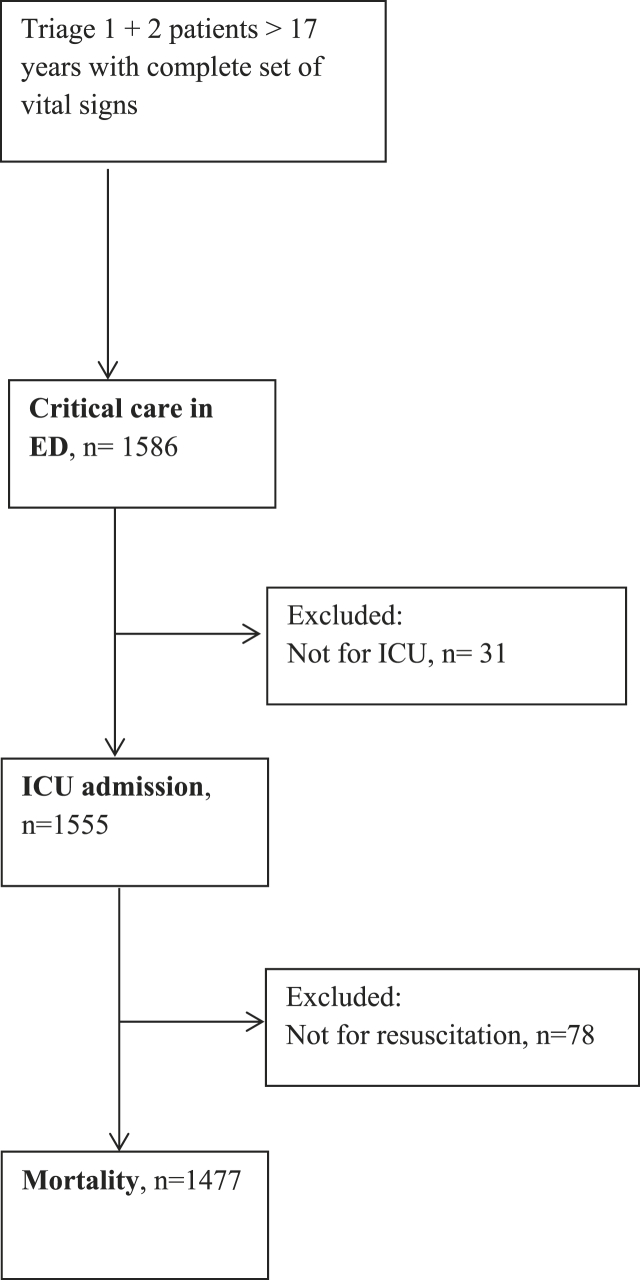
Flowchart of included and excluded patients.

Altogether 1294 triage 1 and 1426 triage 2 patients were eligible for inclusion. Only patients with complete registration of vital signs needed to calculate NEWS2 and those 18 years and older were included (n ​= ​1586). For the outcome ‘mortality’ we excluded patients with a ‘Not for ICU’ and ‘Not for resuscitation’-order (n ​= ​78). For ‘ICU admission’ we excluded patients with a ‘Not for ICU’-order (n ​= ​31) (Fig. 1).

### Outcomes and variables

The primary outcome ‘mortality’ was defined as 30 day mortality and hospital mortality after 30 days. ‘ICU admission’ was defined as admission from ED to the Medical Intensive Care Unit, the Coronary Intensive Care Unit or any other ICU. ‘Critical care in ED’ was defined as any of intubation, other airway interventions, non-invasive ventilation, arterial line, central venous line, pacing, cardioversion, cardiopulmonary resuscitation, pleural catheter or administration of blood products, sedatives, anesthetic agents, antiarrhythmics or vasopressors.^18^ All outcome variables were dichotomous.

NEWS2 (Table 1) was calculated based on vital signs at arrival. The SpO2 scale 1 was used due to no data on hypercapnic respiratory failure.^8^ NEWS2 was used both as a continuous variable and dichotomous with cut-offs >6p, >5p, >4p and >3p. The single-parameter system, hereafter called OUH-criteria, consists of cut-offs for vital signs as well as symptoms and clinical concern. For this study we only used the vital signs criteria (Table 1). The presence of any criteria on arrival was used as a dichotomous variable.

Comorbidity included Charlson Comorbidity Index (CCI)^19^ and history of substance abuse and/or psychiatric illness. CCI was categorized as 0p, 1-2p, 3-4p and >4p,^20^ the latter was dichotomous.

Categories of presenting complaint were made by grouping the most frequent complaints. Discharge diagnosis was based on main ICD-10 diagnosis, and grouped as the categories for presenting complaint. Any diagnosis of infection was categorized as infection, irrespective of site.

### Statistical analysis

IBM SPSS® version 25.0 for Windows (Armonk, NY, USA) and R software for statistical computing^21^ were used for analysis. Categorical variables are presented as number (n) and percentage and continuous as median with interquartile range (IQR). Group-comparison was two-sided, using Chi-square test for categorical and Mann-Whitney rank sum test for continuous variables.

The area under the receiver-operating characteristic curve was used to assess the ability to predict the outcomes. Area under the curve (AUC) of 0.5–0.69 was considered poor, 0.7–0.79 acceptable, 0.8–0.89 excellent and 0.9–1.0 outstanding.^22^

Associations with the outcomes were analyzed using multiple logistic regression. Models were built based on clinical rational (supplement 1). We adjusted for gender, age and CCI for all outcomes. For ‘mortality’ we also adjusted for substance abuse or psychiatric history and critical care or team in ED or ICU admission. For ‘ICU admission’ we adjusted for critical care or team in ED, and for ‘critical care in ED’ for team. We used Hosmer-Lemeshow test to assess goodness of-fit. Data are presented as unadjusted and adjusted odds ratio (OR) with confidence intervals (CI) and p-values.

For all analysis a p<0.05 was regarded statistically significant.

### Ethics

The study was approved by the Data Protection officer at OUH (2016/10319). Since treatment was not affected and data from the registers were anonymous, informed consent was waived.

## Results

Altogether 1586 patients, of which 804 (51%) were triage 1 and had a team response, were included (Table 2). Median age was 63 years and 847 (53%) patients were male. Median NEWS2 at arrival was 6, and 653 (41%) patients were scored ​> ​6. OUH-criteria were present in 497 (31%) patients. A total of 595 (38%) patients received critical care interventions in the ED, 566 (36%) were admitted to ICU and 154 (10%) were dead at 30 days or during hospital stay. Non-survivors were older and with higher CCI than survivors, and fewer had a history of substance abuse and/or psychiatric illness (all p<0.001). More non-survivors had triage 1, OUH-criteria present at arrival, higher NEWS2 and critical care in ED, as well as more Not for resuscitation-orders, compared to survivors (all p<0.001). The most frequent discharge diagnoses were infection (36% in non-survivors, 20% in survivors) and cardiac/circulatory (33% and 29%).

**Table 2:** Characteristics of patients.

	Whole cohort (n ​= ​1586)	Non-survivors (n ​= ​154)	Survivors (n ​= ​1432)	P-value
Age, median (IQR)	63 (32)	83 (14)	61 (32)	<0.001
Male gender	847 (53%)	75 (49%)	772 (54%)	0.22
Charlson Comorbidity Index (n ​= ​148 ​+ ​1369)				<0.001
0p	653 (43%)	30 (20%)	623 (46%)	
1-2p	615 (41%)	64 (43%)	551 (40%)	
3-4p	189 (13%)	34 (23%)	155 (11%)	
>4p	60 (4%)	20 (14%)	40 (3%)	
History of substance abuse and/or psychiatric illness	352 (22%)	14 (9%)	338 (24%)	<0.001
Presenting complaint (n ​= ​151 ​+ ​1383)				<0.001
Cardiac/circulatory	380 (24%)	21 (14%)	359 (26%)	
Acute poisoning	222 (14%)	2 (1%)	220 (16%)	
Respiratory	285 (18%)	44 (29%)	241 (17%)	
Consciousness/neurologic	291 (18%)	36 (24%)	255 (18%)	
Abdominal	52 (3%)	6 (4%)	46 (3%)	
Infection	230 (15%)	32 (21%)	198 (14%)	
Other	74 (5%)	10 (7%)	64 (5%)	
OUH-criteria present at arrival	497 (31%)	79 (51%)	418 (29%)	<0.001
NEWS2 points at arrival				
median (IQR)	6 (5)	9 (5)	5 (6)	<0.001
>6	653 (41%)	112 (73%)	541 (38%)	<0.001
>5	808 (51%)	124 (81%)	684 (48%)	<0.001
>4	938 (59%)	134 (87%)	804 (56%)	<0.001
>3	1077 (68%)	138 (90%)	939 (66%)	<0.001
Triage 1/team response	804 (51%)	116 (75%)	688 (48%)	<0.001
Critical care in ED	595 (38%)	81 (53%)	514 (36%)	<0.001
Not for resuscitation	93 (6%)	52 (34%)	41 (3%)	<0.001
ICU admission	566 (36%)	56 (36%)	510 (36%)	0.85
Primary discharge diagnosis (n ​= ​154 ​+ ​1428)				<0.001
Cardiac/circulatory	460 (29%)	50 (33%)	410 (29%)	
Poisoning	244 (15%)	2 (1%)	242 (17%)	
Respiratory	112 (7%)	12 (8%)	100 (7%)	
Neurologic	87 (6%)	2 (1%)	85 (6%)	
Abdominal	129 (8%)	12 (8%)	117 (8%)	
Infection	343 (22%)	56 (36%)	287 (20%)	
Others	207 (13%)	20 (13%)	187 (13%)	

IQR: interquartile range, OUH: Oslo University Hospital, NEWS2: National Early Warning Score 2, ED: Emergency Department, ICU: Intensive Care Unit.

### NEWS2-scale

**Fig. 2:**
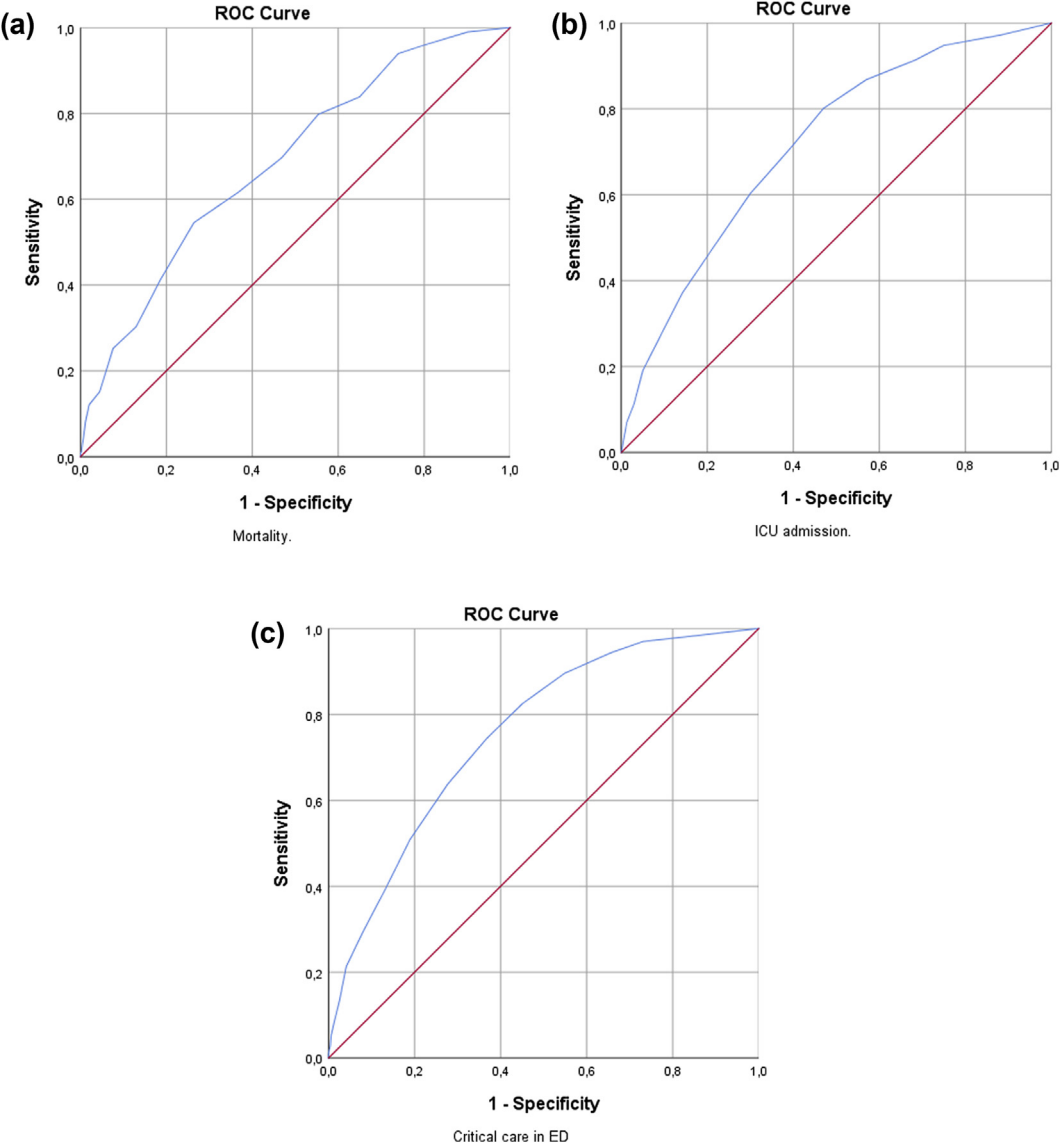
Receiver operator characteristics curve (ROC) for the NEWS2-scale for the outcomes ‘mortality’ (a), ‘ICU admission’ (b) and ‘critical care in ED’ (c).

The NEWS2 scale showed poor prediction of ‘mortality’ (AUC 0.686, CI 0.633–0.739), and acceptable prediction of ‘ICU admission’ (AUC 0.716, CI 0.690–0.742) and ‘critical care in ED’ (AUC 0.756, CI 0.732–0.780) (Table 3). It was associated with all outcomes (all p<0.001). According to ROC-curves the optimal cut-off for ‘mortality’ was 7, for ‘ICU admission’ 4 and for ‘critical care in ED’ 5 (Fig. 2).

**Table 3:** Prediction of and association with outcomes for NEWS2.

	Mortality^a^	ICU admission^b^	Critical care in ED^c^
AUC (CI)	0.686 (0.633–0.739)∗∗	0.716 (0.690–0.742)∗∗	0.756 (0.732–0.780)∗∗
Crude OR	1.203 (1.139–1.272)∗∗	1.252 (1.211–1.293)∗∗	1.318 (1.273–1.364)∗∗
Adjusted OR	1.139 (1.063–1.220)∗∗	1.142 (1.097–1.189)∗∗	1.177 (1.128–1.228)∗∗
Goodness of fit	0.635	0.254	0.827

AUC: Area under the curve, CI: Confidence interval, OR: odds ratio, ICU: Intensive Care Unit, ED: Emergency Department, ∗∗p<0.001.

a OR adjusted for gender, age, Charlson Comorbidity Index (CCI), substance abuse or psychiatric history and critical care or team in ED or ICU admission.

b OR adjusted for gender, age, CCI and critical care or team in ED.

c OR adjusted for gender, age, CCI and team.

### NEWS2 cut-offs and OUH-criteria

#### Mortality

NEWS2 > 6 had the highest AUC for the outcome ‘mortality’ (AUC 0.624, CI 0.567–0.682), which was significantly better than the OUH-criteria (Table 4). All NEWS2 cut-offs and the OUH-criteria had poor prediction with AUC <0.7. Association with the outcome was found for NEWS2 > 6 and the OUH-criteria (both p<0.05); the latter had the highest OR (OR 1.688, CI 1.052–2.708).

**Table 4:** Prediction of and association with outcomes for different cut-offs of NEWS2 and OUH-criteria.

	Mortality^a^	ICU admission^b^	Critical care in ED^c^
NEWS2 > 6			
AUC (CI)	0.624 (0.567–0.682)∗∗	0.651 (0.623–0.680)∗∗	0.680 (0.653–0.708)∗∗
Crude OR	2.766 (1.818–4.209)∗∗	3.542 (2.850–4.402)∗∗	4.591 (3.694–5.707)∗∗
Adjusted OR	1.641 (1.010–2.666)	1.991 (1.522–2.603)∗∗	2.257 (1.721–2.961)∗∗
	p ​= ​0.045∗		
Goodness of fit	0.426	0.057	0.869

NEWS2 ​> ​5			
AUC (CI)	0.614 (0.559–0.669)∗∗	0.657 (0.629–0.686)∗∗	0.688 (0.661–0.715)∗∗
Crude OR	2.606 (1.676–4.053)∗∗	3.740 (2.993–4.673)∗∗	4.999 (3.992–6.259)∗∗
Adjusted OR	1.452 (0.868–2.430)	1.911 (1.450–2.519)∗∗	2.361 (1.782–3.129)∗∗
	p ​= ​0.156		
Goodness of fit	0.847	0.050	0.685

NEWS2 ​> ​4			
AUC (CI)	0.622 (0.570–0.675)∗∗	0.666 (0.638–0.693)∗∗	0.687 (0.661–0.713)∗∗
Crude OR	3.184 (1.927–5.260)∗∗	4.540 (3.561–5.788)∗∗	5.746 (4.494–7.347)∗∗
Adjusted OR	1.766 (0.999–3.122)	2.339 (1.742–3.141)∗∗	2.520 (1.863–3.407)∗∗
	p ​= ​0.050		
Goodness of fit	0.204	0.246	0.646

NEWS2 ​> ​3			
AUC (CI)	0.594 (0.542–0.647)∗	0.649 (0.622–0.676)∗∗	0.673 (0.647–0.700)∗∗
Crude OR	2.800 (1.621–4.835)∗∗	4.963 (3.766–6.542)∗∗	7.064 (5.279–9.452)∗∗
Adjusted OR	1.485 (0.809–2.726)	2.160 (1.558–2.994)∗∗	2.620 (1.862–3.687)∗∗
	p ​= ​0.202		
Goodness of fit	0.756	0.118	0.609

OUH-criteria			
AUC (CI)	0.568 (0.508–0.628)∗	0.651 (0.622–0.680)∗∗	0.685 (0.657–0.713)∗∗
Crude OR	1.821 (1.202–2.758)	4.008 (3.194–5.029)∗∗	5.653 (4.491–7.115)∗∗
	p ​= ​0.005∗		
Adjusted OR	1.688 (1.052–2.708)	1.842 (1.413–2.401)∗∗	3.267 (2.490–4.286)∗∗
	p ​= ​0.030∗		
Goodness of fit	0.474	0.352	0.028

Comparisons of AUCs represented by p-values			
OUH vs NEWS ​> ​6	0.01∗	1.00	0.58
OUH vs NEWS ​> ​5	0.02∗	0.52	0.74
OUH vs NEWS ​> ​4	0.01∗	0.11	0.82
OUH vs NEWS ​> ​3	0.18	0.83	0.18

NEWS2: National Early Warning Score 2, AUC: area under the curve, CI: Confidence interval, OR: odds ratio, vs: versus, ICU: Intensive Care Unit, ED: Emergency Department, ∗p<0.05, ∗∗p<0.001.

a OR adjusted for gender, age, CCI, substance abuse or psychiatric history and critical care or team in ED or ICU admission.

b OR adjusted for gender, age, CCI and critical care or team in ED.

c OR adjusted for gender, age, CCI and team.

#### ICU admission

NEWS2 > 4 had the highest AUC for ‘ICU admission’ (0.666, CI 0.638–0.693), but not significantly better than the OUH-criteria. All NEWS2 cut-offs and OUH-criteria had poor prediction. All NEWS2 cut-offs and the OUH-criteria were associated with the outcome (all p<0.001), with highest OR for NEWS2 ​> ​4 (OR 2.339, CI 1.742–3.141).

#### Critical care in ED

All NEWS2 cut-offs and the OUH-criteria also had poor prediction of ‘critical care in ED’. NEWS2 > 5 had the highest AUC (0.688, CI 0.661–0.715), but not significantly better than the OUH-criteria. All NEWS2 cut-offs and the OUH-criteria were associated with the outcome (all p<0.001). The OUH-criteria had the highest OR of 3.267 (CI 2.490–4.286).

Additional analyses are shown in supplement 2.

## Discussion

The NEWS2 scale showed poor prediction of ‘mortality’ and adequate prediction of ‘ICU admission’ and ‘critical care in ED’. It was strongly associated with all outcomes. All NEWS2 cut-offs and the OUH-criteria showed poor prediction of all outcomes. The OUH-criteria had the strongest association with ‘critical care in ED’ and ‘mortality’, and NEWS2 > 4 had the strongest association with ‘ICU admission’.

In clinical practice the key interest is to find a trigger to initiate a response, whether it is a NEWS2 cut-off or single-parameter criteria. It should balance between risk of under-triage and over-use of resources. Below we compare our results with NEWS studies from the ED and NEWS2 studies irrespective of setting, due to few studies.

### Mortality

Mortality seems the most used outcome for NEWS and NEWS2 studies. Timeframes varies from 1 day to 6 years; settings from prehospital to in-hospital and populations from general to patients with a variety of diagnosis.^10^^,^^11^^,^^14^, ^15^, ^16^, ^17^^,^^23^, ^24^, ^25^, ^26^, ^27^, ^28^ This complicates comparison of findings.

Prognostic accuracy of NEWS and NEWS2 in the abovementioned studies varies from poor to outstanding. Poor accuracy, as in our study, was found in two ED-studies of NEWS.^14^^,^^28^ Two studies investigating different timeframes found falling AUCs as timeframe expanded.^24^^,^^25^ Outcomes much later in time than the ED-stay have been criticized for not representing acuity of the patient at ED arrival, as factors later in the stay may influence the outcome.^29^ This is supported by the abovementioned studies and our findings of ‘mortality’ having the poorest accuracy of the three outcomes and being the outcome farthest away in time.

Despite poor accuracy, we found the NEWS2 scale to be strongly associated with ‘mortality’ in multivariate analysis. This has also been found in other studies of NEWS, regardless of timeframe.^14^^,^^16^ We found lower OR for ‘mortality’ than the other outcomes, further supporting the use of outcomes closer in time.

NEWS2 > 6 had better accuracy than the OUH-criteria. In multivariate analysis, however, the OUH-criteria had an equally strong association, with a higher OR. Others have found NEWS cut-offs to be more strongly associated with or having higher specificity for mortality than single-parameter systems.^7^^,^^26^ Both studies investigated ward patients and used different single-parameter systems, which may explain the different results from ours.

Given poor accuracy of NEWS2 > 6 and better association of the OUH-criteria, we argue that using this cut-off as a criterion for team response or resuscitation room (RR) management gives no added value for the outcome ‘mortality’ as defined in this study. Mortality should in the future be investigated in a shorter timeframe, we suggest 24 and 48 ​h from arrival.

### ICU admission

Only a few studies investigated the outcome ICU admission. For ICU admission within 24 ​h of in-patients, both Smith et al and Pimentel et al found excellent accuracy of NEWS/NEWS2.^7^^,^^23^ Corfield et al investigated NEWS in ED sepsis-patients, and found adequate prediction of ICU admission within 2 days; more similar to our result.^11^

We found the optimal NEWS2 cut-off to be 4, which also had the strongest association with the highest OR when adjusting for co-factors. Corfield et al found association with ICU admission within 2 days after age-adjustment for the NEWS categories 7–8 points and >8 points, the latter having the strongest association.^11^

Almost one in four in our cohort had a discharge diagnosis of infection. NEWS >4 have been found to be associated with an increased risk of ICU admission or death in sepsis patients,^8^ and the many patients with infection could thus contribute to this cut-off value having the highest association with ICU admission. ICU capacity will also affect admittance, and our results suggest that capacity allows admitting patients with less profound organ-symptoms.

NEWS2 > 4 seem to be more strongly associated with ICU admission than the OUH-criteria, with equal accuracy. We therefore suggest considering this cut-off as an additional criterion for team approach or RR management.

### Critical care in ED

**T**his outcome had the highest AUCs and ORs for both the NEWS2-scale, different NEWS2 cut-offs and the OUH-criteria. Interestingly, for cut-offs of NEWS2 the OR was increasing as NEWS2 was falling. This could be due to many patients having single-organ symptoms, thus limiting amount of NEWS2-points, but still requiring critical care in ED.

This outcome is closest in time from arrival. For our purpose; identification of critically ill medical patients in the ED in order to trigger a response, we argue that the outcomes closest in time is most clinically relevant. Based on the results it seems that ‘critical care in ED’ is the most relevant for our cohort. It reflects potentially life-saving measures needed, which is the reason why clinicians want to trigger a response in the ED. It of course requires ED personnel being able to provide ICU-level of care, which might be challenging due to lack of expertise and ED overcrowding.^1^ For other purposes; i.e. identifying patients at risk of later mortality, but who don’t necessarily need a certain response or resuscitation at ED arrival, investigations of outcomes later in time could have higher relevance. Surprisingly, we found no other studies of NEWS or NEWS2 using ‘critical care in ED’ or similar outcomes.

The OUH-criteria had the highest OR for this outcome, and we see no benefit of adding a NEWS2 cut-off in our criteria to ensure that patients receive critical care in ED by a multidisciplinary team. This is contradictory to the discussion above, where we suggest considering NEWS2 > 4 as an additional criterion due to its association with ICU admission. In other words, it seems that the OUH-criteria ensures that patients receive the critical care needed in the ED by a multidisciplinary team, but that NEWS2 > 4 identifies more patients in need of ICU admission. Addition of NEWS2 > 4 could thus ensure ICU level of care in the ED and a smooth transition without delay from ED to ICU, reported to be missing for many critically ill patients.^1^ We nevertheless believe that NEWS2 plays an important role in monitoring of patients during the ED stay in order to detect deterioration, and it is implemented as an observation tool in our ED.

For this outcome, critical care in ED, it seems that our criteria-based system worked better than the tested scoring-system; NEWS2, raising the question whether EDs need criteria or a score for this specific outcome. The effect of clinical judgement was not included in this study. We however believe that clinical judgement should always accompany any system being used, which is why clinical concern is also a part of the OUH-criteria.

### Limitations

This was a retrospective single-center study, and might not be representative for other cohorts of patients. It can, independent of study design, be difficult to compare results of different studies due to differences in organization and characteristics of different EDs, hospitals and national health care systems.^13^^,^^16^ Confounding was reduced by using multivariate analysis.

A large group of the eligible patients was excluded due to missing data on vital signs needed to calculate NEWS2. Most patients missed one part-score of NEWS2, and Glasgow Coma Scale, respiration rate and temperature were the part-scores most commonly missing (supplement 3), this is in line with previous findings of missing parameters.^30^ Others experiencing missing data on vital signs have either set the values as normal,^16^^,^^17^ used multiple imputation^10^^,^^15^^,^^23^^,^^25^ or excluded patients.^11^^,^^12^^,^^27^ All solutions could result in bias. We found no significant differences in age, gender, comorbidity or critical care given between included and excluded patients(supplement 3).

Due to no data on hypercapnic respiratory failure, we used the SpO2 scale 1 in NEWS2-calculation. This datum is often unavailable during ED triage, and thus reflects clinical reality.^31^ We can nevertheless not add new insight on this major change in NEWS2 compared to NEWS.^8^

‘Mortality’ was a combination of the available in-hospital and 30 day mortality. For reasons already discussed, we recommend investigating mortality within 24/48 ​h in similar future studies.

## Conclusion

The NEWS2-scale showed poor prediction of ‘mortality’ and adequate prediction of ‘ICU admission’ and ‘critical care in the ED’ in critically ill medical patients. It was associated with the same outcomes. NEWS2 > 6 had better prediction than the OUH-criteria for the outcome ‘mortality’, and NEWS2 > 4 had a stronger association with ‘ICU admission’.

For the purpose of identification in order to trigger a response, we argue that the outcomes closest in time are most clinically relevant. As such, performance of the OUH-criteria was acceptable. NEWS2 > 4 should be considered as an additional criterion due to its association with ICU admission. We recommend further research into identification of critically ill medical patients in the ED, to optimize identification and management in the future.

## Credit author statement

Stine Engebretsen: Conceptualization, Methodology, Formal analysis, Writing – original draft

Dag Jacobsen: Conceptualization, Writing – review and editing, Supervision

Stig Tore Bogstrand: Conceptualization, Methodology, Writing – review and editing, Supervision

Valeria Vitelli: Conceptualization, Methodology, Formal analysis, Writing – review and editing

Rune Rimstad: Conceptualization, Methodology, Writing – review and editing, Supervision, Project administration.

## Declaration of competing interest

None conflict of interest.
