# Supplementary material for: NEWS2 versus a single-parameter system to identify critically ill medical patients in the emergency department
Source: Resusc Plus. 2020 Aug 6;3:100020. doi: 10.1016/j.resplu.2020.100020 (PMC8244393; doi:10.1016/j.resplu.2020.100020)
Supplement: Multimedia component 1 [file mmc1.docx]

Supplement 1. Multiple logistic regression models

Legend: Red arrow: timeline from clinical presentation at arrival to outcome (blue boxes). White boxes: Factors potentially influencing clinical presentation or outcome. Blue arrows: direction of influence. Bold writing: factors adjusted for. Italic writing: patients excluded.

Mortality

**Substance abuse or psychiatric history**

Restrictions in care:

*Not for resuscitation, Not for ICU*

**CCI**

**Age**

Clinical presentation at arrival:

OUH-criteria

NEWS2

**Gender**

Outcome: Mortality

**Critical care or team in ED or ICU admission**

ICU admission

Substance abuse or psychiatric history

Restrictions in care:

*Not for ICU*

**CCI**

Outcome: ICU admission

**Age**

**Gender**

Clinical presentation at arrival:

OUH-criteria

NEWS2

**Critical care in ED and/or team**

Critical care in ED

Substance abuse or psychiatric history

**CCI**

**Team**

**Age**

**Gender**

Clinical presentation at arrival:

OUH-criteria

NEWS2

Outcome: Critical care in ED
