# Supplementary material for: NEWS2 versus a single-parameter system to identify critically ill medical patients in the emergency department
Source: Resusc Plus. 2020 Aug 6;3:100020. doi: 10.1016/j.resplu.2020.100020 (PMC8244393; doi:10.1016/j.resplu.2020.100020)
Supplement: Multimedia component 2 [file mmc2.docx]

Supplement 2. Additional analysis

|  | Mortality | ICU admission | Critical care in ED |
| --- | --- | --- | --- |
| NEWS2 > 6  Sensitivity  Specificity  PPV  NPV | 61.6%  63.3%  10.8%  95.8% | 60.2%  70.1%  52.6%  76.1% | 63.7%  72.4%  58%  76.8% |
| NEWS2 > 5  Sensitivity  Specificity  PPV  NPV | 69.7%  53.1%  9.7%  96.1% | 70.7%  60.8%  49.9%  79% | 74.5%  63.2%  54.8%  80.5% |
| NEWS2 > 4  Sensitivity  Specificity  PPV  NPV | 79.8%  44.6%  9.4%  96.9% | 80.1%  53%  48.5%  82.8% | 82.5%  54.9%  52.3%  84% |
| NEWS2 > 3  Sensitivity  Specificity  PPV  NPV | 83.8%  35.1%  8.5%  96.8% | 86.8%  43%  45.7%  85.5% | 89.6%  45.1%  49.5%  87.8% |
| OUH-criteria  Sensitivity  Specificity  PPV  NPV | 42.4%  71.2%  9.6%  94.5% | 50.5%  79.7%  57.9%  74.5% | 54.5%  82.5%  65.2%  75.1% |

PPV: positive predictive value, NPV: negative predictive value
