# Supplementary material for: NEWS2 versus a single-parameter system to identify critically ill medical patients in the emergency department
Source: Resusc Plus. 2020 Aug 6;3:100020. doi: 10.1016/j.resplu.2020.100020 (PMC8244393; doi:10.1016/j.resplu.2020.100020)
Supplement: Multimedia component 3 [file mmc3.docx]

Supplement 3. Characteristics of included and excluded patients

|  | Included  (n=1586) | Excluded  (n=1134) | p-value |
| --- | --- | --- | --- |
| Age, median (IQR) | 63 (32) | 65 (35) | 0.16 |
| Male gender | 847 (53.4%) | 631 (55.6%) | 0.25 |
| Charlson Comorbidity Index (n=1517/1098)  0p  1-2p  3-4p  >4p | 653 (43%)  615 (40.5%)  189 (12.5%)  60 (4%) | 498 (45.4%)  428 (39%)  119 (10.8%)  53 (4.8%) | 0.30 |
| History of substance abuse or psychiatric illness | 352 (22.2%) | 226 (19.9%) | 0.16 |
| Critical care  No critical care  Team or critical care interventions in ED without ICU admission  ICU admission from ED | 612 (38.6%)  408 (25.7%)  566 (35.7%) | 476 (42%)  249 (22%)  409 (36.1%) | 0.06 |
| Number of NEWS2 part-scores missing (n=1134, excluded patients only)  0 (excluded due to age < 18)  1  2  3  4  5  6  7 (all) |  | 10 (0.9%)  756 (66.7%)  222 (19.6%)  83 (7.3%)  17 (1.5%)  4 (0.4%)  1 (0.1%)  41 (3.6%) |  |
| Type of NEWS2 part-score missing (n=1134, excluded patients only)  RR  SpO2  O2 supplement  BP  Pulse  Temperature  GCS |  | 394 (34.7%)  113 (10%)  90 (7.9%)  102 (9%)  102 (9%)  330 (29.1%)  694 (61.2%) |  |

IQR: interquartile range, ED: Emergency Department, ICU: Intensive Care Unit, NEWS2: National Early Warning Score 2, RR: respiration rate, O2: oxygen, BP: blood pressure, GCS: Glasgow Coma Scale
